# The Relationships Among Structural Social Support, Functional Social Support, and Loneliness in Older Adults: Analysis of Regional Differences Based on a Multigroup Structural Equation Model

**DOI:** 10.3389/fpsyg.2021.732173

**Published:** 2021-09-09

**Authors:** Haifeng Li, Cong Wang

**Affiliations:** ^1^School of Psychology, Fujian Normal University, Fuzhou, China; ^2^Fuzhou Hualun Middle School, Fuzhou, China

**Keywords:** structural social support, functional social support, loneliness, regional difference, older adults

## Abstract

**Objective:** This study investigated the relationship between structural social support and loneliness and explored whether functional social support had an intermediate role therein. It also employed a multigroup structural equation model to compare mediation models among older adults living in cities, towns, and rural areas.

**Methods:** Using a self-made demographics questionnaire, the structural-functional social support scale, and the 3-item UCLA loneliness scale, this study collected information from 1,325 older adults identified *via* convenient sampling.

**Results:** The results showed that as: (1) compared with older adults living in towns, older urban, and rural adults had higher structural social support and experienced less loneliness, while older adults’ functional social support showed no difference among the three regions (2) An analysis of the models of regional differences indicated that functional social support served as a full mediator in the relationship between structural social support and loneliness in urban older adults, and a partial mediator for older adults living in towns and rural areas.

**Conclusion:** The relationship between structural social support and loneliness is mediated by functional social support, and this mediation model varies between older adults in cities and towns/rural areas. This study helps us understand possible mechanisms through which structural social support impacts loneliness. It suggests that nursing strategies for older adults should be adjusted according to the region and direct greater focus on the function (or quality) of the social support network and older adults living in towns.

## Introduction

Loneliness refers to situations in which a person feels distressed, depression, and disengagement due to a lack of social or emotional life (Killeen, 1998). Loneliness in older adults is associated with a decline in body function and an increase in mortality (Cacioppo et al., 2002; Holwerda et al., 2012; Perissinotto et al., 2012). It is also closely related to the level of depression, psychological distress, and anxiety (Paul et al., 2006; Golden et al., 2009; Cacioppo et al., 2015).

Social support is a factor protecting against loneliness (Golden et al., 2009; Chen et al., 2014; Chen and Feeley, 2014; Zhang and Silverstein, 2020). It can be evaluated from both structural (i.e., quantity) and functional (i.e., quality) aspects. Structural support^1^ refers to the existence and quantity of social relationships within an individual’s social network (Sherbourne and Stewart, 1991). The density and size of one’s social network and frequency of social contact can be used as indicators of structural support (Stokes, 1985; Green et al., 2001; Heylen, 2010; Gallo et al., 2015). Functional support is conceptualized as one’s subjective assessment of the adequacy of their relationships or the quality of their social relationships (House, 1987; Hittner and Swickert, 2001; Santini et al., 2015). Sometimes, functional support is measured by one’s satisfaction with personal relationships or support from others (Murphy et al., 2000; Davidson et al., 2016).

The convoy model of social relations provides one theoretical basis for this study to investigate the impact of social support on loneliness. According to the convoy model, individuals are surrounded by supportive others who vary in their closeness, e.g., family members, other relatives, friends, neighbors, and co-workers (Antonucci et al., 2014). These members not only constitute a support network for older adults but also provide older adults with many kinds of social support (e.g., aid, affect, and affirmation exchanges). Therefore, many studies have shown that both structural and functional supports can effectively alleviate loneliness in older adults (Sherbourne and Stewart, 1991; Green et al., 2001; Pinquart and Sorensen, 2001; Cheng et al., 2010; Heylen, 2010; de Jong Gierveld et al., 2015; Kemperman et al., 2019; Wittenborn et al., 2020). For example, a meta-analysis of 149 articles published from 1948 to 1999 found that both the quantity and quality of social networks were closely related to loneliness (Pinquart and Sorensen, 2001). Data from 1,414 adults over age 55 in Belgian showed that both the structural (as measured by contact frequency with friends, family, and acquaintances living outside the household and the number of good friends) and functional (as measured by satisfaction with each of the personal social contacts) social relationships significantly affected social loneliness (Heylen, 2010). A study on nursing home residents in Hong Kong found that the frequency of contact and functional support (as measured by confiding, showing affection, and advice and guidance from family, other relatives, friends, and staff and fellow residents) were associated with loneliness (Cheng et al., 2010). Another study of 3,799 respondents over age 65 in Canada found that social network size and composition and satisfaction with network contacts were found to be related to loneliness (de Jong Gierveld et al., 2015). Therefore, this study proposed the hypothesis (a): Structural and functional supports were negatively associated with loneliness.

In addition, according to the convoy model, it is clearly necessary to have some quantity of relationships if one is to have high-quality relationships (Antonucci et al., 2014). Hence, it can be predicted that individuals with higher structural support are more likely to have higher functional support. Montes-Berges and Augusto (2007) used Vaux’s subjective social support scale to measure the functional support of nurses and the objective social support scale to measure the density of their social networks. The authors found that there was a moderate correlation between the two aspects of social support (*r*=0.42). Using the 12-item interpersonal support evaluation list and the social network index to assess functional and structural support, respectively, researchers found that the association between structural and functional support measures was positive and moderate in magnitude (*r*=0.28; Gallo et al., 2015). In addition, research on the social networks of entrepreneurs also found that the frequent contacts with members of their social network significantly correlated with the quality of the relational interaction (*r*=0.31; Pollack et al., 2016). Therefore, this study proposed the hypothesis (b): Structural support significantly correlates with functional support.

Another theoretical basis for this study is the theory of socioemotional selectivity which predicts that with increasing age, people attach more importance to the quality of relationships (Carstensen, 1995). Accordingly, many studies have shown that functional support has a stronger prediction on loneliness than does structural support (e.g., Pinquart and Sorensen, 2001; Routasalo et al., 2006; Hawkley et al., 2008; Antonucci et al., 2014). Despite both being related to loneliness, the quality of social network correlated more strongly with loneliness than did the quantity (Pinquart and Sorensen, 2001). Loneliness in older adults was found to be closely associated with expectations of and satisfaction with contacts with children and friends, but not with the frequency of these contacts (Routasalo et al., 2006). Another study found that when controlling for the influence of demographic information, such as gender, age, and ethnicity, satisfaction with one’s social network had a stronger prediction on the loneliness of older adults than did the social network size (Hawkley et al., 2008).

In conclusion, a higher level of structural support can predict a higher level of functional support, and functional support predicts loneliness better than structural support does, so it is speculated that functional support may serve as an intermediary factor between structural support and loneliness. Empirical research has shown that functional support can be used as an intermediary, affecting individuals’ depression and loneliness. For example, Fiori et al. (2006) used the perceived quality of social relations as an internal mechanism to explore its mediating role between social support type and depression. The results indicated that perceived quality of social relations partially mediated the association between network type and older adults’ depressive symptomatology. Another study focused on the risk factors of loneliness in older adults, finding that satisfaction with their social relations partially mediated the association between the number of social relations and social loneliness (Heylen, 2010). Therefore, this study proposed the hypothesis (c): Functional support mediates the relationship between structural support and loneliness.

According to the life course theory, social change has a significant impact on personal life and development (Elder et al., 2003). The development of urbanization leads to different politics, economies, cultures, and environments in different regions. Therefore, researchers have also paid close attention to the regional differences in social support and loneliness among older adults (e.g., Paykel et al., 2003; Wang and Zhou, 2010; Baernholdt et al., 2012; Su et al., 2015; Abel et al., 2016; Gao et al., 2020). The regional differences of loneliness are different between China and other countries. In other countries, many studies have found that rural older adults reported a lower level of loneliness than did urban or urban-cluster older adults (Abel et al., 2016 in Uganda; Kaleigh et al., 2019 in the United States; Paul et al., 2019 in New Zealand), or urban older adults (25.3%) had a higher proportion of “lonely” (the score of 3-item UCLA loneliness scale greater than 6 was classified as “lonely”) than older adults living in town/fringe (21%) and rural (23.1%) areas (Victor and Pikhartova, 2020 in England), although two studies in Finnish have shown the opposite results (Savikko et al., 2005; Routasalo et al., 2006). However, in China, a study has found that living in a rural (as opposed to urban) area is a specific factor to the Chinese context and is associated with a higher level of loneliness (Yang and Victor, 2008). Therefore, studies in China have consistently shown that rural older adults experienced more loneliness than did urban older adults (e.g., Wang and Zhou, 2010; Wei, 2012; Su et al., 2015).

When it comes to social support, fewer studies investigated the regional differences of structural and functional supports. Therefore, the structural or functional support referred to below does not completely correspond to the definitions of structural or functional support in the current study. Studies in the United Kingdom found that older adults in semi-rural and rural areas participated in more social activities and had stronger network structures (except in terms of friends) than did those in urban areas (Bowling et al., 1995; Paykel et al., 2003). Similarly, Baernholdt et al. (2012) found that older adults in rural areas had a larger family support network but a smaller friend and religious support network than did those in rural-urban adjacent and urban areas. However, a study conducted in Iowa found that there were no differences in the size of social network, the frequency of social interaction, the amount of instrumental support, and the subjective level of social support between urban and rural older adults (Evans, 2009).

In China, many studies used the social support rating scale to measure social support from objective and subjective dimensions (Xiao, 1994). Objective support measures an individual’s living arrangement and sources of social support, whereas subjective support refers to an individual’s emotional experience and satisfaction of being respected and supported. It is measured by the closeness with family members, friends, and neighbors. Although objective support and structural support, and subjective support and functional support differ in their definitions and are measured in different ways, we can roughly regard objective support as structural support and subjective support as functional support. Most studies have found that compared with rural older adults, urban older adults had a higher level of objective (structural) support and subjective (functional) support (e.g., Wang et al., 2016; Mao et al., 2017; Gao et al., 2020). However, some studies found that objective (structural) support of rural older adults was significantly higher than that of urban older adults (Zeng, 2006; Li et al., 2013), and subjective (functional) support had no difference between urban and rural older adults (Zeng, 2006).

Because of the inconsistency of definitions and measurement tools, it is hard to draw a unified conclusion on the regional differences of structural and functional supports in China. In addition, the way some studies simply divided older adults into urban and rural groups may weaken the conclusion of these studies. In China, to facilitate administrative management, regions are divided into three categories: provincial, municipal, and county/town level (The Central People’s Government of the People’s Republic of China, 2005). Rural areas are generally subordinate to towns. Thus, some studies classified older adults who were investigated in towns and rural areas into a single group as rural older adults (Zeng, 2006; Wang and Zhou, 2010; Li et al., 2013; Wang et al., 2016). However, the last two decades of urbanization in China have led to differences in rural, town, and urban politics, economies, cultures, and environments. There may be differences in social support and loneliness between older adults in towns and rural areas. Therefore, the differences in structural support, functional support, and loneliness among older adults in cities, towns, and rural areas in China are still unclear. Nevertheless, according to the previous literature, this study still proposed the hypothesis (d): compared with older adults living in towns and rural areas, urban older adults reported less loneliness, and the hypothesis (e): compared with older adults living in towns and rural areas, urban older adults had higher structural and functional supports. Based on the hypotheses of this study, after testing the mediating effect of functional support, this study will further explore the regional differences of this mediating effect.

In sum, this study explores the mediating role of functional support in the relationship between structural support and loneliness in older adults. To explore the regional differences of this mediating effect, we used a multigroup structural equation model (SEM) to compare the models among older adults in cities, towns, and rural areas.

## Materials and Methods

### Participants

This study was approved by the Ethics Committee of the School of Psychology at Fujian Normal University. We employed summer college students to collect data. A group of 1,424 older adults were collected through convenient sampling. Participants were recruited from 11 provinces or province-level municipalities in China, including Anhui, Beijing, Fujian, Gansu, Guangdong, Guangxi, Guizhou, Henan, Shanxi, Yunnan, and Zhejiang. All participants were asked to sign an informed consent form and then complete a questionnaire and face-to-face interview. Individuals had to: (1) be aged≥60years, (2) have no missing data on their questionnaires, except those requesting demographic information, (3) have no contradictory answers (e.g., choosing “widowed” on marital status but “living only with spouse” on living arrangement), and (4) have no obvious regular answers (e.g., choosing the same option for 10 or more successive questions). Of the total, 99 participants were excluded, thereby making the effective rate as high as 93%. The remaining 1,325 participants were distributed among cities, towns, and rural areas, with a mean age of 69.27years (*SD*=6.92; age range=60–97years); 46.6% were male. Some participants did not disclose some of their demographic information, such as age (*N*=2), gender (*N*=6), education level (*N*=2), living arrangement (*N*=7), and economic satisfaction (*N*=1).

### Measures

Structural-functional social support scale: Almquist et al. (2017) were the first to extract nine items from two dimensions of the interview schedule for social interaction (ISSI; Henderson et al., 1980) to measure structural and functional social supports. The four items of structural support were taken from the availability of social integration on the ISSI. These included as: (1) How many people who share your interests do you know and have contact with? (2) How many people do you know that you meet or talk to during a week? (3) How many friends do you have who can visit you in your home and feel “at home”? and (4) How many people can you speak openly with? For each of these items, the response options were as: (1) none, (2) 1–2, (3) 3–5, (4) 6–10, (5) 11–15, and (6) more than 15. Higher mean scores indicated more structural support or a larger social network. The five items of functional support were extracted from the availability of attachment on the ISSI. These included as: (1) There is someone special who I really feel supports me, (2) There is someone special who is close to me, (3) Others appreciate what I do for them, (4) There are people around me who I can easily ask for favors, and (5) There are other persons outside my family that are close to me and that I can turn to in times of hardship. The response options for each item were as: (1) disagree completely, (2) disagree, (3) agree, and (4) agree completely. The higher the mean score, the higher the functional support the people perceived. Functional support in this study was measured on five levels, with scores ranging from 1 to 5. A “Neither agree nor disagree” option was set in the middle to make the choices more in line with the Chinese cultural background (Si and Cullen, 1998; Lee et al., 2002; Zhao et al., 2017). To ensure the validity of these items, this study used Amos 22.0 to construct a two-factor SEM for confirmatory factor analysis, which yielded the following outputs: $\chi_{51}^{2}$=185.807, *df*=26, *p*<0.001, $\chi_{51}^{2}$/*df*=7.147, TLI=0.939, CFI=0.956, PCFI=0.690, and RMSEA=0.068 (CI_90_=0.059, 0.078), indicating the model was acceptable (Hu and Bentler, 1999). The Cronbach’s α of structural and functional supports in this study was 0.774 and 0.813, respectively.

The 3-item UCLA loneliness scale: compiled by Hughes et al. (2004), the 3-item UCLA loneliness scale contains only three items: (1) How often do you feel that you lack companionship? (2) How often do you feel left out? and (3) How often do you feel isolated from others? Participants respond regarding the frequency of the above experiences. The options include as: (1) hardly ever (2) some of the time, and (3) often. Loneliness scores are between 3 and 9. The higher the score, the stronger the loneliness felt. In this study, the Cronbach’s α for the scale was 0.799.

### Statistical Analysis

All analyses were conducted using the SPSS 19.0 and AMOS 22.0. Frequencies and cross-tabulations gave the distribution of socio-demographic variables, while means and standard deviations showed the scores of structural support, functional support, and loneliness in older adults in cities, towns, and rural areas. A Pearson correlation was used to establish correlations, while a one-way ANOVA was employed to explore the regional differences among these study variables. A SEM with latent variables was used to evaluate whether functional support mediated the relationship between structural support and loneliness. Then, a SEM with a multigroup analysis was used to assess the regional differences of the mediation model among older adults in cities, towns, and rural areas.

In this study, the maximum likelihood method estimation was used in the SEM analyses. A preliminary analysis of the distribution of data showed that the absolute value of the skewness coefficient for each item was between 0.311 and 0.869, and the kurtosis coefficient was between 0.149 and 0.859. When the skewness is less than 2 and the kurtosis is less than 7, the maximum likelihood method estimation is robust (Finney and DiStefano, 2006). For the SEM, the *χ*^2^ statistic is usually significant in large sample studies, often causing researchers to reject appropriate models that should be accepted (Kenny, 2015). Therefore, this study used other fit indices, including the comparative fit index (CFI), Tucker-Lewis index (TLI), and approximate root mean square error of approximation (RMSEA). A good model fit is achieved if the CFI and TLI values are above 0.90 and the RMSEA value ranges from 0.05 to 0.08, providing a reasonable and appropriate fit (Kline, 2010). For the multigroup SEM, Akaike’s information criterion (AIC) and the expected cross-validation index (ECVI) were used to verify the measurement invariance across models in different regions. When multiple models in the results are fit, the model with the smallest AIC and ECVI values is the most suitable (Wu, 2010).

## Results

### Descriptive Statistics

The data on the socio-demographics of participants by region are presented in Table 1. The mean ages of the different groups and total sample were all around 69years, and nearly half of the total sample was female. For education level, about half (51.4%) of the total had primary school and below, with 27.3 and 21.2% having had secondary and high school (and above), respectively. Older adults in rural areas had a greater proportion (68.1%) of primary school and below than did older adults living in cities (40.5%) and towns (33.9%). The majority of the sample (75.6%) was married, regardless of residence type. In terms of economic condition, 38.9% of the total sample rated their economic condition as “Satisfied” or “Very satisfied,” 15.4% rated it as “Dissatisfied” or “Very dissatisfied,” and 45.6% rated it as “Average.” Among the three regions, a higher proportion of urban older adults reported satisfaction with their economic condition (51.9%) than did older adults in towns and rural areas (around 33% of older adults for both areas).

**Table 1:** Demographic characteristics of older adults in cities, towns, and rural areas.

Socio-demographics	City (*N*=393; 29.7%)	Town (*N*=330; 24.9%)	Rural area (*N*=602; 45.4%)	Total (*N*=1,325; 100%)
Age (*M*±*SD*)	69.57±7.47	69.18±6.62	69.12±6.72	69.27±6.92
Female	202 (51.4%)	168 (50.9)	331 (55.0%)	701 (52.9%)
**Education level** ^*^				
Primary school and below	159 (40.5%)	112 (33.9%)	410 (68.1%)	681 (51.4%)
Secondary school	104 (26.5%)	131 (39.7%)	126 (20.9%)	361 (27.3%)
High school and above	129 (32.8%)	86 (26.1%)	66 (11.0%)	281 (21.2%)
Married (including remarriage)	299 (76.1%)	242 (73.3%)	460 (76.4%)	1001 (75.6%)
**Economic satisfaction** ^*^				
Very dissatisfied	7 (1.8%)	9 (2.7%)	15 (2.5%)	31 (2.3%)
Dissatisfied	32 (8.1%)	49 (14.9%)	92 (15.3%)	173 (13.1%)
Average	150 (38.2%)	164 (49.7%)	290 (48.2%)	604 (45.6%)
Satisfied	168 (42.8%)	95 (28.8%)	179 (29.6%)	442 (33.3%)
Very satisfied	36 (9.2%)	13 (3.9%)	26 (4.3%)	75 (5.7%)

* *indicates a significant difference (p<0.001) among older adults in cities, towns, and rural areas, based on a chi-squared test; percentages may not add up to 100 due to missing data*.

Table 2 shows the values of structural support, functional support, and loneliness in older adults in cities, towns, and rural areas. A one-way ANOVA showed that there were significant differences in the structural support of older adults among different regions, *F*=5.145, *p*=0.006. A post-hoc analysis using the Bonferroni test indicated that the level of structural support for the town-based older adults was significantly lower than that of rural (*p*=0.007) and urban (*p*=0.027) older adults, but there was no significant difference between rural and urban older adults. Moreover, there was no significant difference in the functional support among the older adults dwelling in all three regions. Loneliness for each group differed significantly, *F*=30.694, *p*<0.001. A post-hoc analysis indicated that older adults in cities experienced less loneliness than did older adults in towns (*p*<0.001) and rural (*p*<0.001) areas, and older adults in rural areas experienced less loneliness than did older adults in towns (*p*=0.031).

**Table 2:** Structural social support, functional social support, and loneliness of older adults by region.

Variable	City (*N*=393)	Town (*N*=330)	Rural area (*N*=602)	*F*	*p*
Structural social support (0–5)	3.33±0.97	3.15±0.83	3.35±0.98	5.145	0.006
Functional social support (1–5)	3.67±0.63	3.60±0.60	3.63±0.61	1.494	0.225
Loneliness (3–9)	4.47±1.63	5.38±1.61	5.09±1.66	30.694	< 0.001

### Bivariate Correlations

Table 3 presents Spearman’s correlations among the study variables. The results showed that structural and functional supports were positively correlated with one another, and both were negatively correlated with loneliness. The results of a Fisher r-to-z transformation indicated that the correlation between structural support and loneliness in urban older adults was significantly lower than that for older adults in towns and rural areas (*Zr*=2.06, *p*=0.039).

**Table 3:** Correlations among structural social support, functional social support, and loneliness in older adults by region.

Variable	Structural social support	Functional social support
Structural social support	1	
Functional social support	0.35^**^ (0.37^**^, 0.39^**^, 0.32^**^)	1
Loneliness	−0.22^**^ (−0.13^*^, −0.28^**^, −0.24^**^)	−0.30^**^ (−0.22^**^, −0.33^**^, −0.32^**^)

*Values outside the parenthesis represent the correlation coefficients of the whole sample; values inside the parenthesis represent the correlation coefficients of the samples from cities, towns, and rural areas*.

^*^p< 0.05

*and*

** *p< 0.01.*

### Mediation Analysis of Functional Support

A SEM with mediation pathways was created to evaluate whether functional support represented mechanisms through which structural support might impact loneliness among older adults; this was accomplished by performing bootstrapping to calculate 95% bias-corrected confidence intervals for indirect effects.

**Figure 1:**
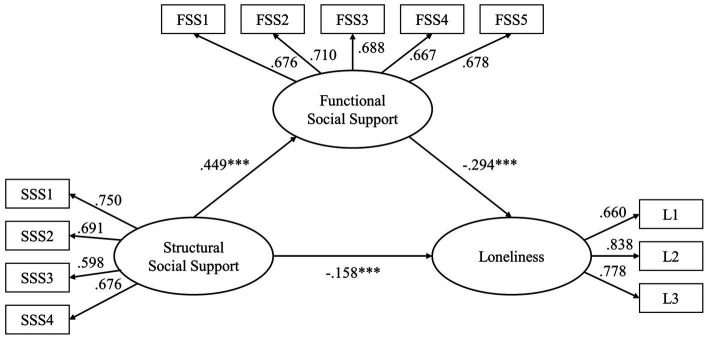
Mediation model showing the relationship between structural social support and loneliness as mediated by functional social support. SSS, structural social support; FSS, functional social support; and ^***^ *p*<0.001.

The result showed that the total effect of structural support on loneliness (standardized total effect=−0.290, *p*<0.001) was significant. An indirect pathway existed between structural support and loneliness *via* functional support (standardized indirect effect=−0.132, *p*=0.005). The direct effect of structural support on loneliness (standardized direct effect=−0.158, *p*=0.005) was also significant in the mediation model, indicating that functional support partially mediated the relationship between structural support and loneliness. The model fit indices indicated an acceptable fit, $\chi_{51}^{2}$=241.939, *p*<0.001, CFI=0.962, TLI=0.951, and RMSEA=0.053 (CI_90_=0.047, 0.060). The size of the indirect effect *via* functional support was 83.54% (−0.132/−0.158) of that direct effect. For more detail, see Figure 1.

### Multigroup SEM: Measurement Invariance

The regional differences of relationships among structural support, functional support, and loneliness were examined using SEM with a multigroup analysis. First, measurement invariance was verified by two models that gradually added constraints. Model 1 was a configural model with free paths between each group. The model fit indices indicated an adequate fit, *χ*^2^=372.952, *df*=153, CFI=0.957, TLI=0.944, RMSEA=0.033 (CI_90_=0.029, 0.037), AIC=606.952, and ECVI=0.459. Model 2 was a measurement weights model with equal factor loadings on all groups. The model fit indices were also acceptable, *χ*^2^=401.398, *df*=171, CFI=0.955, TLI=0.947, RMSEA=0.032 (CI_90_=0.028, 0.036), AIC=599.398, and ECVI=0.453. Comparisons across models showed that the increase in chi-squared values (Δ*χ*^2^=28.446, *p*=0.056) was not statistically significant and changes in other model fit indices were small, indicating robust measurement consistency across the groups in cities, towns, and rural areas used in this research. In this study, the model with the smaller AIC and ECVI values, i.e., the equal measurement weights model (Model 2), was used as the more suitable model in the subsequent multigroup analysis.

### Multigroup Comparison

**Figure 2:**
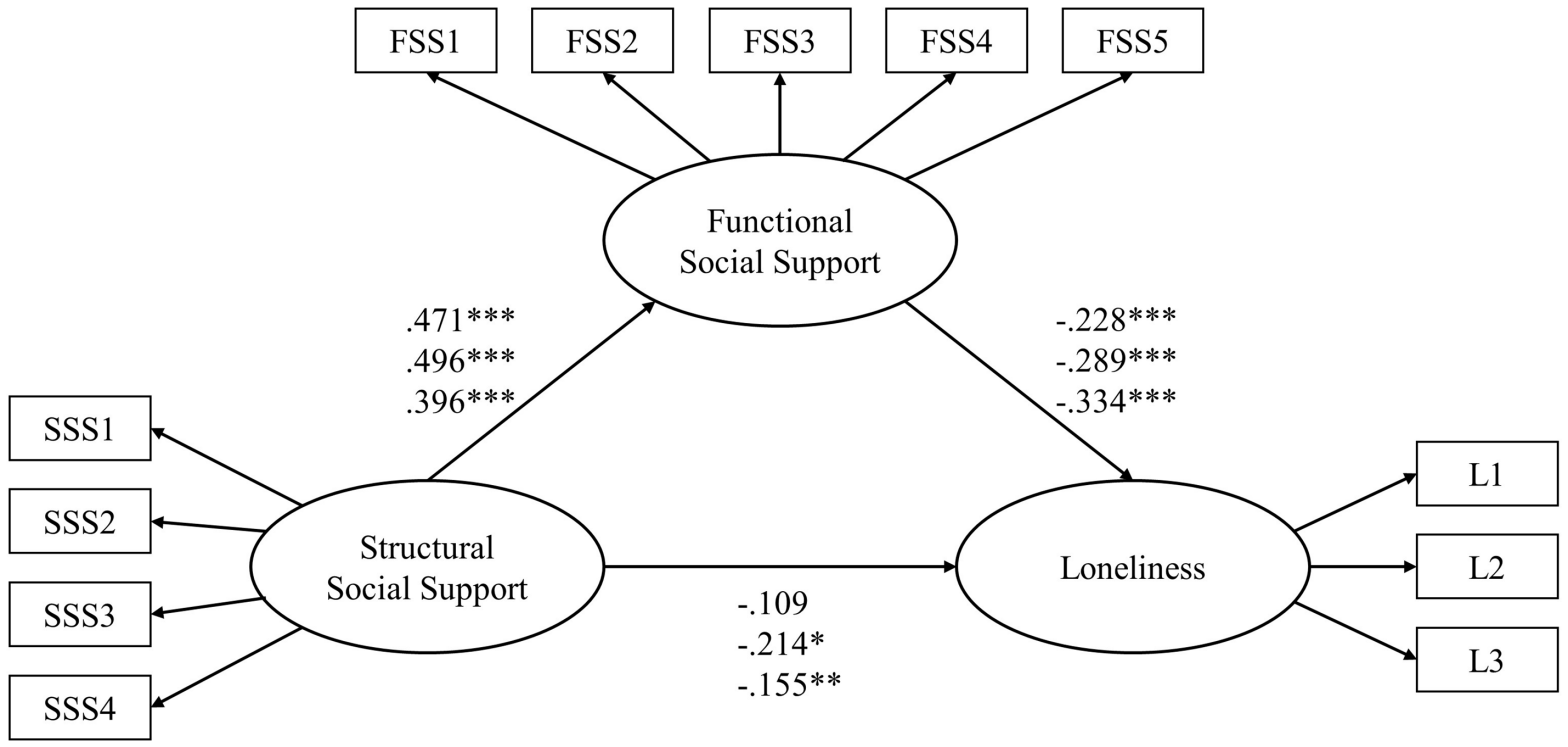
Mediation models for older adults in cities, towns, and rural areas. The three values from top to bottom on each path represent parameters corresponding to the models for older adults in cities, towns, and rural areas. SSS, structural social support; FSS, functional social support; and ^*^p<0.05, ^**^p<0.01, and ^***^p<0.001.

According to residence place, the sample was divided into urban, town-based, and rural groups. The path coefficients of each model were compared by critical ratios for differences (CRD) between parameters to explore regional differences in the mediating effect of functional support (see Table 4; Figure 2). If the CRD between parameters is greater than 1.96, the two parameters are significantly different (Jang and Kim, 2018). The results showed that as: (1) Functional support fully mediated the relationship between structural support and loneliness in urban older adults. The size of the indirect effect *via* functional support was 98.17% (−0.107/−0.109) of that direct effect. However, (2) functional support partially mediated the impact of structural support on loneliness in older adults residing in towns and rural areas. The mediation effect accounted for 66.81 and 85.16% of the total effect, respectively. (3) Comparing the path coefficients of the three models, it was determined that structural support for older adults in towns had a greater prediction on functional support than did the same support for rural older adults.

**Table 4:** Standardized path coefficients in SEM for older adults in cities, towns, and rural areas.

Path	Path coefficient		CRD
	City	Town	
SSS→FSS (total effect)	0.471^***^	0.496^***^	0.964
FSS→Loneliness	−0.228^**^	−0.289^**^	−0.582
SSS→Loneliness (direct effect)	−0.109	−0.214^*^	−1.386
SSS→FSS→Loneliness (indirect effect)	−0.107^**^	−0.143^**^	
	**Town**	**Rural Areas**	
SSS→FSS (total effect)	0.496^***^	0.396^***^	−2.086
FSS→Loneliness	−0.289^**^	−0.334^**^	−0.532
SSS→Loneliness (direct effect)	−0.214^**^	−0.155^**^	0.847
SSS→FSS→Loneliness (indirect effect)	−0.143^**^	−0.132^***^	
	**City**	**Rural Areas**	
SSS→FSS (total effect)	0.471^***^	0.396^***^	−1.221
FSS→Loneliness	−0.228^**^	−0.334^**^	−1.272
SSS→Loneliness (direct effect)	−0.109	−0.155^**^	−0.825
SSS→FSS→Loneliness (indirect effect)	−0.107^**^	−0.132^***^	

*SSS, structural social support; FSS, functional social support; and CRD, critical ratios for differences between parameters*.

* *p< 0.05*;

** *p< 0.01*

*and*

*** *p< 0.001*.

## Discussion

The purpose of this study was to examine the impact of structural support on loneliness and the mediation role of functional support in this relationship. Multigroup analysis was used to compare the mediation models among older adults in cities, towns, and rural areas. The results showed that as: (1) Structural support for older adults in urban and rural areas was higher than for older adults in towns. Functional support showed no significant differences among older adults in the three regions. Loneliness in urban older adults was significantly less than in older adults in towns and rural areas, and loneliness in rural older adults was significantly less than in older adults in towns. (2) In general, the impact of structural support on the loneliness of older adults was partially achieved through functional support. The mediation effect accounted for 83.54% of the total effect. (3) There are regional differences in the mediating effect of functional support. Specifically, functional support for urban older adults fully mediated the relationship between structural support and loneliness. The mediation effect accounted for 98.17% of the total effect. However, functional support for older adults in towns and rural areas partially mediated the impact of structural support on loneliness. The mediation effect accounted for 66.81 and 85.16% of the total effect, respectively.

This study found that urban older adults experienced less loneliness than did older adults in towns and rural areas, a conclusion that supported the hypothesis (d). This result is consistent with the results found in the previous studies (e.g., Wang and Zhou, 2010; Wei, 2012; Su et al., 2015). The novel finding was rural older adults experienced less loneliness than did older adults in towns (the following explanation for the regional difference in structural support can also explain this result). However, the regional differences of social support did not fully validate the hypothesis (e). Structural support for older adults in cities, towns, and rural areas showed a V-shaped relationship that urban and rural older adults had higher structural support than older adults in towns, whereas functional support had no significant differences among the three groups. For the result of lower structural support in older adults in towns, we tried to explain it from the basis of social support in different regions. The social support in rural areas tends to be based on blood relationships, whereas the social support in urban areas tends to be based on professional relationships (Zhu and Shao, 2005). However, due to the influence of urbanization, the blood relationships had been destroyed, while the professional relationships had not been fully established in towns (Cai, 2005; Li et al., 2012). Therefore, the structural support for older adults in towns was lower than for urban and rural older adults. A recent study classifying respondents (who are not limited to older adults) as living in cities, towns, or rural areas found the same V-shaped relationship that compared with urban and rural residents, residents living in towns reported less social support (Wang et al., 2015), supporting the above explanation. However, inconsistent with the hypothesis (e), functional support had no differences among older adults in the three regions. This result is consistent with the prediction of socioemotional selectivity theory that older adults tend to pay more attention to more intimate and satisfying relationships than they do when they are younger (Carstensen, 1995). Although most previous studies have found that subjective support of urban older adults is higher than that of rural older adults (e.g., Wang et al., 2016; Mao et al., 2017; Gao et al., 2020), a few studies also have found that urban and rural older adults have no significant difference in subjective support (Zeng, 2006) or satisfaction with support (Xu et al., 2018). However, no previous studies have investigated the regional differences in the perspective of functional support in Chinese older adults. Due to the different definitions between functional support and subjective support, it is difficult to conclude whether this result reflects the actual situation in China. Future research is needed to replicate this result.

As for the relationship between social support and loneliness, this study found that functional support played a partial mediating role in the impact of structural support on loneliness. This result not only verified our hypotheses (a, b, and c) but also supported the prediction of the convoy model of social relations. As previous studies have shown, the size, composition, and frequency of social support can affect loneliness (e.g., Cheng et al., 2010; de Jong Gierveld et al., 2015; Kemperman et al., 2019; Wittenborn et al., 2020). Therefore, structural support can directly predict loneliness in older adults. Moreover, structural support can indirectly impact loneliness *via* functional support. Higher structural support suggests that older adults may have multiple social roles in their social network and therefore can obtain more and higher-quality social connectedness and integration (Moen, 2001). Older adults can also gain a sense of belonging from their social identity (Dutton et al., 1994), eventually reducing their loneliness.

Furthermore, this study found the regional differences of the mediating effect. Specifically, functional support for urban older adults fully mediated, and for town-based and rural older adults partially mediated, the impact of structural support on loneliness. This result can be explained by social support for older adults in different regions relying on different social groups. Researchers have found that rural residents are more likely to seek support from spouses and relatives, while urban residents are more likely to seek support from friends and colleagues (Cai et al., 1997; Zhu and Shao, 2005). Despite changes stemming from reform and opening up, China’s rural economy still considers the family to be the basic unit of social production. The strong economic relationship between family members collaborating in production increases their dependence on one another in all aspects of their daily lives (Cai et al., 1997). Many generations of rural families in China still live together under the same roof. Such a large-scale, long-term, and stable social network can ensure that rural older adults have both a satisfactory quantity and quality of social support, jointly reducing their loneliness. Compared with the similarities found between older adults in cities and towns, older adults in towns and rural areas are closer in terms of living conditions, such as employment opportunities, income levels, and living standards. Therefore, the impact pattern of social support on loneliness is similar for older adults in towns and rural areas.

However, unlike households in towns and rural areas, urban households have lost the function of production. Urban residents are more inclined to obtain social support from professional ties (such as colleagues and friends; Fang and Hu, 2003; Zhu and Shao, 2005). This suggests that urban residents’ social support is easily affected by occupational changes. Compared with residents in towns and rural areas, urban residents, especially urban youth, are more unstable in terms of their employment and change their addresses more frequently (He, 1991). Therefore, the social networks of urban older adults are unstable and their social support structures are often destroyed. Faced with such situations, urban older adults often take the initiative, accepting and adjusting to their social networks and focusing their time and energy on cultivating higher-quality social support. Therefore, only when their social support based on professional ties is of high quality is it possible to effectively relieve urban older adults’ loneliness.

In addition, through a multigroup SEM, as compared with rural older adults, the structural support for older adults in towns was found to predict their functional support better. In other words, the same amount of structural support can provide older adults in towns with more psychological satisfaction than what would be experienced by rural older adults. The perceived discrepancy hypothesis of loneliness based on cognitive theory proposes that the degree of loneliness depends not only on actual social relations but also on the individual’s expectations of these relations (Perlman and Peplau, 1998). That is, loneliness is a subjective feeling that occurs when there is a discrepancy between individuals’ actual and expected social relations. To avoid loneliness and minimize this perceived discrepancy, people can not only modify their expectations on social relations but also achieve sufficient social support to balance the two (Burholt et al., 2017). According to the results of the present study, compared to rural older adults, older adults in towns not only experienced more loneliness but also had less structural support. In the face of this unfavorable situation, older adults in towns may actively adjust their expectations to reduce the discrepancy and give full value to everyone in their social network to meet their needs. Therefore, as opposed to rural older adults, the structural support of older adults in towns predicted their functional social support better. Of course, this inference needs to be verified in future research.

Several limitations on this study should be considered. First, our results do not support causal relationships among structural support, functional support, and loneliness due to the cross-sectional design. However, there may be a causal or mutual causal relationship between structural and functional supports. The large social network of older adults will lead to high-quality social relations, which further expand their social network, and vice versa. Further research could eliminate this limitation by adopting a longitudinal design to explore the casual relationship and replicate our findings. Second, the participants of this study were recruited from cities in 11 provinces with different politics, economies, cultures, and environments. Therefore, older adults from these cities may have different levels of social support and loneliness. However, the sample size of this study is not large enough to support comparing these differences among different cities. Future research could increase samples to investigate the impact of this factor on social support and loneliness of older adults. Finally, the sample was recruited using convenient sampling, indicating that most of the participants were physically healthy. Therefore, the generalizability of our results to China and other societies remains to be investigated.

In conclusion, the current study demonstrates that functional support plays a mediating role in the relationship between structural support and loneliness. The importance of this study is the division of older adults into three categories, according to their place of residence. This work shows that older adults from different regions have different levels of structural support and loneliness, and the mediation model is different between older adults in cities and towns/rural areas. This study will help researchers better understand how different types of social support interact to decrease loneliness in older adults, suggesting that nursing strategies for older adults in different regions should be focused differently, with the emphasis on the function of social support and on older adults living in towns.

## Data Availability Statement

The original contributions presented in the study are included in the article/Supplementary Material, and further inquiries can be directed to the corresponding author.

## Ethics Statement

The studies involving human participants were reviewed and approved by the School of Psychology, Fujian Normal University. The patients/participants provided their written informed consent to participate in this study.

## Author Contributions

HL conceived and designed the survey. CW collected the data. CW and HL analyzed the data and wrote the paper. All authors contributed to the article and approved the submitted version.

## Funding

This work was supported by grants from the National Social Science Foundation of China (16CSH047) and the Social Science Planning Project of Fujian Province (FJ2018C033).

## Conflict of Interest

All the authors declared that the research was conducted in the absence of any commercial or financial relationships that could be construed as a potential conflict of interest.

## Publisher’s Note

All claims expressed in this article are solely those of the authors and do not necessarily represent those of their affiliated organizations, or those of the publisher, the editors and the reviewers. Any product that may be evaluated in this article, or claim that may be made by its manufacturer, is not guaranteed or endorsed by the publisher.

## Footnotes

^1^For simplicity, structural social support and functional social support are referred to as structural support and functional support, respectively.
